# Ursolic Acid Inhibits the Initiation, Progression of Prostate Cancer and Prolongs the Survival of TRAMP Mice by Modulating Pro-Inflammatory Pathways

**DOI:** 10.1371/journal.pone.0032476

**Published:** 2012-03-12

**Authors:** Muthu K. Shanmugam, Tina H. Ong, Alan Prem Kumar, Chang K. Lun, Paul C. Ho, Peter T. H. Wong, Kam M. Hui, Gautam Sethi

**Affiliations:** 1 Department of Pharmacology, Yong Loo Lin School of Medicine, National University of Singapore, Singapore, Singapore; 2 Division of Cellular and Molecular Research, Humphrey Oei Institute of Cancer Research, National Cancer Centre, Singapore, Singapore; 3 Cancer Science Institute of Singapore, National University of Singapore, Singapore, Singapore; 4 School of Anatomy and Human Biology, The University of Western Australia, Crawley, Perth, Western Australia, Australia; 5 Department of Pharmacy, National University of Singapore, Singapore, Singapore; Mizoram University, India

## Abstract

Prostate cancer is one of the leading causes of cancer death among men worldwide. In this study, using transgenic adenocarcinoma of mouse prostate (TRAMP) mice, the effect of diet enriched with 1% w/w ursolic acid (UA) was investigated to evaluate the stage specific chemopreventive activity against prostate cancer. We found that TRAMP mice fed with UA diet for 8 weeks (weeks 4 to 12) delayed formation of prostate intraepithelial neoplasia (PIN). Similarly, mice fed with UA diet for 6 weeks (weeks 12 to 18) inhibited progression of PIN to adenocarcinoma as determined by hematoxylin and eosin staining. Finally, TRAMP mice fed with UA diet for 12 weeks (weeks 24 to 36) demonstrated markedly reduced tumor growth without any significant effects on total body weight and prolonged overall survival. With respect to the molecular mechanism, we found that UA down-regulated activation of various pro-inflammatory mediators including, NF-κB, STAT3, AKT and IKKα/β phosphorylation in the dorsolateral prostate (DLP) tissues that correlated with the reduction in serum levels of TNF-α and IL-6. In addition, UA significantly down-regulated the expression levels of cyclin D1 and COX-2 but up-regulated the levels of caspase-3 as revealed by immunohistochemical analysis of tumor tissue sections. Finally, UA was detected in serum samples obtained from various mice groups fed with enriched diet in nanogram quantity indicating that it is well absorbed in the GI tract. Overall, our findings provide strong evidence that UA can be an excellent agent for both the prevention and treatment of prostate cancer.

## Introduction

Prostate cancer is the second leading cause of cancer-related death among men in Western countries after lung cancer [1]. Chronic inflammation is increasingly being recognized as a mediator for many cancers [2], [3], [4] and considerable evidence suggests that it plays a major role both in the development and progression of prostate cancer [5]. First, patients with symptomatic prostatitis are more susceptible to developing prostate cancer. Second, prostate cancer has been associated with sexually transmitted infections. Third, decreased risk of prostate cancer is linked with increased intake of fruits and vegetables, antioxidants, and non-steroidal anti-inflammatory drugs (NSAID) [6], [7]. Fourth, normal prostate undergoes proliferative inflammatory atrophy (PIA) before forming prostate intraepithelial neoplasia (PIN), the precursor of prostate cancer [8]. Fifth, PIA can overexpress inflammatory enzyme COX-2 [9]. Sixth, the transcription factors NF-κB and STAT3, both major mediator of inflammation, are constitutively active in prostate cancer tissues [10]. And seventh, NF-κB-regulated inflammatory cytokines such as interleukin (IL)-6 is an autocrine growth factor known to be secreted by prostate cancer tissues [11], [12]. Therefore, it is reasonable to suggest that agents that can suppress inflammatory mediators have a potential for both the prevention and treatment of prostate cancer.

UA (3β-hydroxy-urs-12-en-28-oic acid), a pentacyclic triterpenoid derived from berries, leaves, flowers, and fruits of medicinal plants, such as *Rosemarinus officinalis*, *Eriobotrya japonica*, *Calluna vulgaris*, *Ocimum sanctum*, and *Eugenia jumbolana* is one such agent that has been extensively studied for its anti-inflammatory and anticancer activities in the past decade [13]. UA has been reported to suppress the proliferation of a variety of tumor cells, to induce apoptosis, and to inhibit tumor promotion, metastasis, and angiogenesis [14], [15], [16], [17]. Our group is currently investigating the unexplored potential of UA for the prevention and treatment of prostate cancer and has recently reported in two separate studies that UA can indeed suppress the growth of prostate xenograft in nude mice and also inhibit distant site metastasis by modulating the CXCR4/CXCL12 signaling cascade [18], [19].

The transgenic adenocarcinoma of mouse prostate (TRAMP) model of prostate cancer is based on a transgene consisting of probasin (PB) promoter-driven SV40 T antigen (Tag) expression. This transgenic mouse is preferable to implantation models because in this model prostate cancer spontaneously develops through a series of well-defined stages. They exhibit remarkable similarities to human prostate cancer progression from the PIN stage to invasive adenocarcinoma that metastasizes to liver, lung and GI tract via activation of a transgene that is hormonally regulated by androgens [20], [21]. Moreover, all TRAMP mice develop well-differentiated carcinoma (WDC) between 8–12 weeks of age and metastases to distant sites between 18 and 24 weeks of age [22]. In the present study, we investigated the potential effects of UA enriched diet in preventing the development, progression as well as overall survival of tumors in TRAMP mice. Our data clearly indicates for the first time that feeding mice with a diet rich in UA can significantly inhibit the progression from PIN to adenocarcinoma, and tumor growth. It can also prolong the survival of mice through the modulation of multiple signaling cascades.

## Results

**Figure 1:**
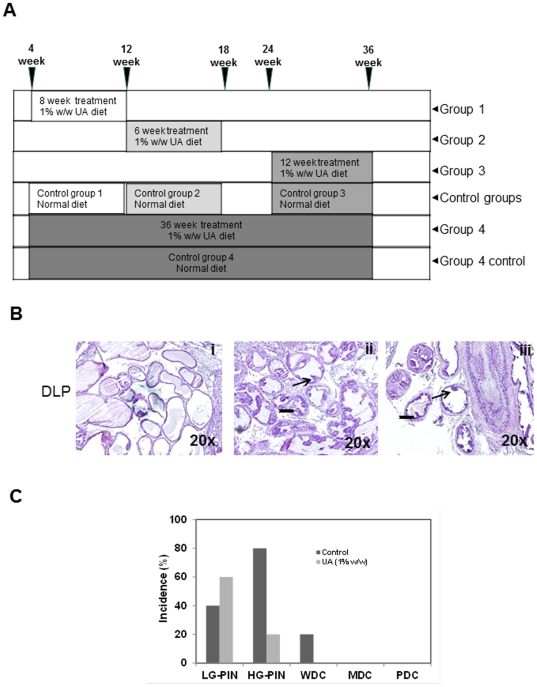
Schematic diagram showing the experimental design to study the stage specific effects of UA treatment on prostate tumor progression. **A**, Male TRAMP-mice 4 weeks of age were fed with UA enriched diet for 8 weeks (Group 1); 12 weeks old mice for 6 weeks (Group 2); and 24 weeks old mice for 12 week (Group 3). Mice were sacrificed at the end of the treatment periods. Age-matched control TRAMP-mice received normal diet for the same time periods. For survival studies, Groups 1 to 3 were similarly treated while Group 4 was fed with UA enriched diet for 36 weeks starting at 4 weeks of age. **B**, H&E stained sections of DLP of non-TRAMP prostate (i), Group 1 control (ii), and UA-treated (iii) TRAMP prostate. **C**, Bar chart shows the incidence of LG-PIN, HG-PIN, WDC, MDC and PDC in DLP of control and Group 1 mice. A slight increase in LG-PIN and a decrease in HG-PIN were observed (n = 5) after UA treatment. No MDC and PDC were observed in Group 1 and corresponding controls. Arrow indicates LG-PIN; notched right arrow indicates HG-PIN.

The present study was designed to investigate the potential effect(s) of UA on early tumor development, tumor progression and the overall survival of TRAMP mice (Fig. 1A).

### UA suppresses prostatic intraepithelial neoplasia (PIN) formation

It has been reported previously that TRAMP mice exhibit increased epithelial stratification in the dorsolateral prostate, epithelial cells with variably elongated nuclei with condensed chromatin with flat patterns of low-grade (LG) PIN (LG-PIN) by 4–8 weeks of age and tufted micropapillary and a cribriform pattern of HG-PIN by 6–10 week of age [22].To investigate if UA suppresses PIN formation, 4-week-old mice were fed with UA (1%, w/w) containing diet for 8 weeks and sacrificed at 12 weeks of age (Group 1). The wet weight of the prostate with seminal vesicles did not appreciably change in both the control and UA fed TRAMP mice (data not shown). Visual examination of the abdominal cavity also did not reveal unusual enlargement of the seminal vesicles, prostatic lobes or pelvic lymph nodes. Consumption of UA enriched diet was well tolerated without evidence of toxicity in terms of animal appearance, behavior and body weight.

While non-TRAMP mice did not show any PIN formation (Fig. 1B, i), TRAMP mice animals showed LG- and HG-PIN and well differentiated carcinoma (WDC) (Fig. 1B, ii and iii). The effects of UA-treatment on the formation of PIN are shown in Fig. 1C. LG-PIN was seen in 60% of UA-treated mice compared to 40% in controls while HG-PIN was seen in 20% of UA-treated mice as opposed to 80% in controls. This demonstrated that UA treatment had reduced the formation of PIN as well as delayed the progression from LG- to HG-PIN. In addition, only one animal showed moderately (MDC) or poorly differentiated carcinoma (PDC) at this stage. These data strongly suggest that UA has the potential to be used as a chemopreventive agent.

### UA prevents the progression from PIN to prostate cancer

**Figure 2:**
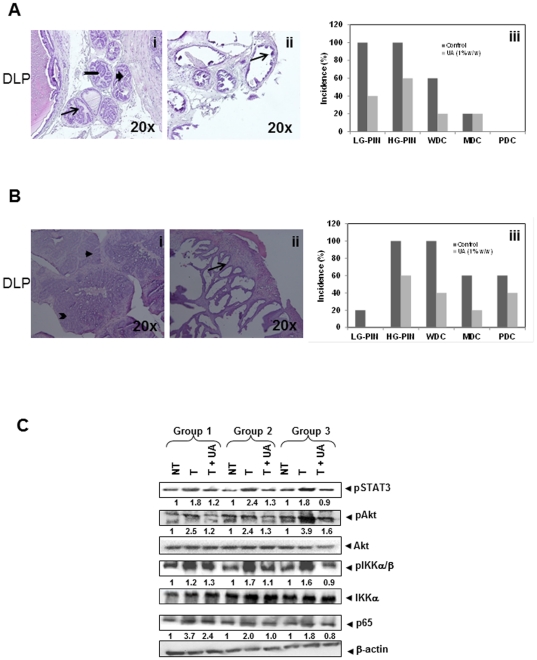
Hematoxylin and Eosin stained dorso lateral prostate of TRAMP mice. **A**, H&E stained section of DLP of Group 2 control (i) and UA-treated (ii) TRAMP prostate. Arrow indicates LG-PIN, notched right arrow indicates HG-PIN, and block right arrow indicates WDC. (iii) Bar chart shows the incidence of LG-PIN, HG-PIN, WDC, MDC and PDC in DLP of control and Group 2 mice. Reductions in PIN, WDC and MDC were observed after UA treatment. **B**, H&E stained section of DLP of Group 3 control (i) and UA-treated (ii) TRAMP prostate. As described in A; Arrow head indicates MDC and chevron indicates PDC. **C**, Western blot of pSTAT3, pAKT, pIKKα/β, and p65 proteins in DLP in Groups 1, 2 and 3 mice. DLP lysates was prepared and immunoblotting was performed as described in Materials and Methods. Equal protein loading for pAKT and pIKKα/β was determined by stripping and probing for total AKT and IKKα, p65 and STAT3 blots were stripped and reprobed for β-actin to determine equal protein loading. The band density was quantitated using Image J software. NT = non-TRAMP-mice; T = TRAMP controls; T+UA = UA-treated TRAMP-mice.

To determine whether or not UA can prevent progression from PIN to prostate cancer, 12-weeks-old TRAMP-mice were fed with control or UA (1%, w/w) enriched diet for 6 weeks and then sacrificed at 18 weeks (Fig. 1A, Group 2). At 18 weeks of age, the DLP of TRAMP mice exhibited higher incidence of LG-PIN, HG-PIN, WDC and MDC (Fig. 2A, i & ii) when compared to those observed at younger ages (Fig. 1B & C). No significant changes in appearance, body weight and food consumption were observed between Group 2 mice and their controls. However, there was an appreciable but statistically insignificant decrease in the wet weight of prostate gland with the seminal vesicles in UA fed mice (data not shown). UA-treatment reduced the incidence of LG-PIN by 60%, HG-PIN by 40% and WDC by 40% (Fig. 2A, iii).Overall, there was a noticeable shift towards the higher incidences of non-cancerous DLP in UA-treated mice.

### UA suppresses the growth of established prostate cancer *in vivo*

To determine if UA can suppress the growth of established prostate cancer, 24-week-old TRAMP-mice were fed with a control or UA (1%, w/w) enriched diet for 12 weeks and then sacrificed at the 36 weeks (Fig. 1A, Group 3). At this age, visual examination of the abdominal cavity clearly revealed enlarged seminal vesicles, DLP and other prostate lobes in the control TRAMP-mice. The DLP showed higher incidence of WDC, MDC and PDC when compared to those observed at younger ages, suggesting that a majority of the animals had cancerous lesions in the DLP (Fig. 2B, i & ii). In contrast, UA–treated TRAMP mice showed reduced incidence of HG-PIN by 40%, WDC by 60%, MDC by 40%, and PDC by 20% (Fig. 2B iii). Overall, the results indicated that UA-treated mice showed non-cancerous lesions ranging from LG-PIN and HG-PIN to normal stroma compared to 100% incidence of prostate cancer in control TRAMP mice.

### UA inhibits the activation of AKT, IKKα/β, NF-κB and STAT3 in DLP of TRAMP mice

We have recently reported that UA can inhibit AKT, IKKα/β, NF-κB and STAT3 activation in androgen dependent and independent prostate cancer cell lines [18]. In this study, we further examined the effects of UA enriched diet on the activation of pro-inflammatory mediators in the DLP of TRAMP mice (Fig. 1A). As shown in Fig. 2C, progressive increase in the phospho (p) -AKT, -pIKKα/β, pSTAT3, and p65 expression were observed from Group 1 to Group 3 TRAMP mice as the cancer gradually progressed from LG-PIN formation in group 1 at 4–12 week of age, HG-PIN and WD carcinoma in DLP in group 2 at 12–18 week of age, and WDC, MDC and PDC at 24–36 week of age. Age-matched non-TRAMP C57BL/6 mice, used as negative controls, did not show any change in the levels of these proinflammatory proteins. Interestingly, we observed that UA treatment resulted in approximately 40–50% inhibition in the expression of these phosphorylated proteins in the DLP of group 2 and group 3 as compared to untreated TRAMP mice, while only a slight decrease was observed in group 1 (Fig. 2C). The levels of the non-phosphorylated AKT and IKKα were minimally affected by UA-treatment (Fig. 2C). Taken together, our data clearly indicated that the suppression of pro-inflammatory AKT, NF-κB and STAT3 activation plays a role in the inhibition of the progression of prostate cancer in TRAMP mice by UA.

### UA inhibits serum TNF-α and IL-6 levels in TRAMP mice

**Figure 3:**
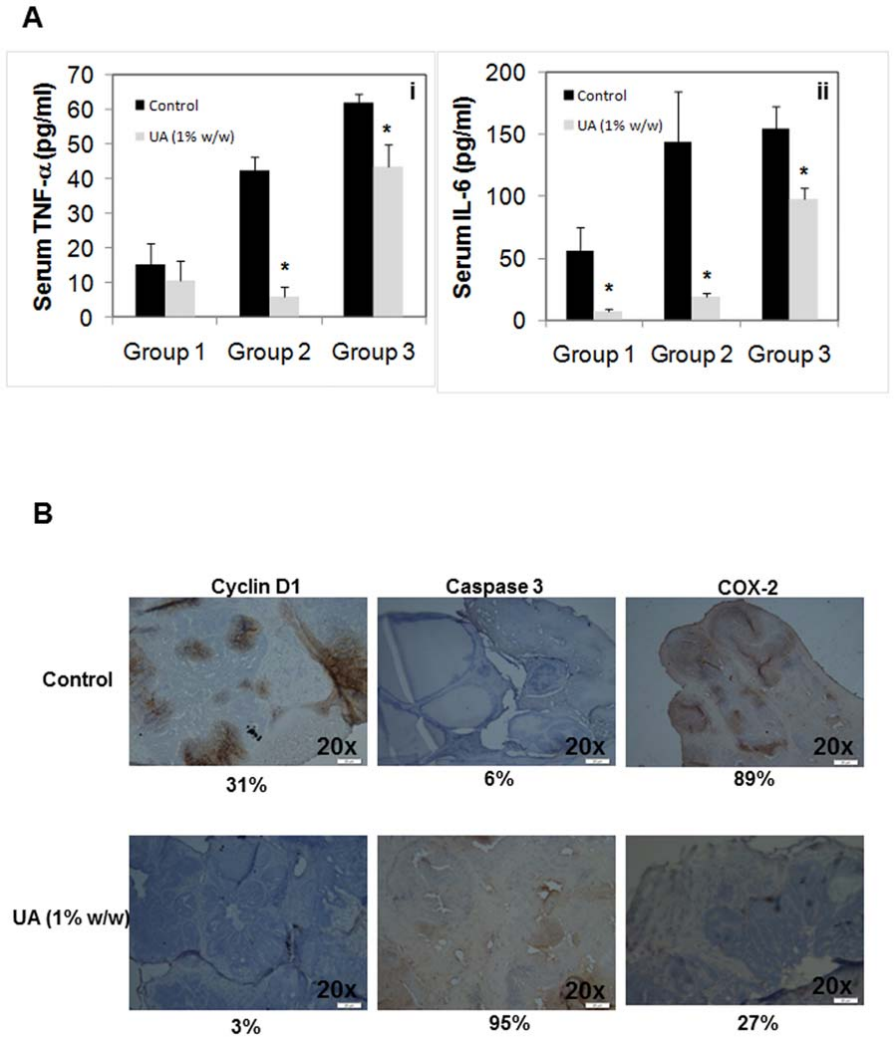
Enzyme linked immunosorbent assay. **A.** Suppression of serum TNF-α (i) and IL-6 (ii) by UA-treatment. Groups 1–3 mice were treated as described in Materials and Methods. Sandwich ELISA assay was performed as per manufacturers' instruction (R&D systems, USA) to determine the levels of TNF-α and IL-6. *Statistical significance (P<0.05). **B,** Immunohistochemical analysis of cyclin D1, caspase 3 and COX-2 in tumor tissues in Group 3 TRAMP mice. DLP tumor tissues, embedded in paraffin blocks, were cut into 5 µM tissue sections and probed for cyclin D1, caspase 3 and COX-2 immunoreactivities as described in the Materials and Methods. UA-treatment (1% w/w) decreased the expression of cyclin D1 and COX-2 but increased caspase-3 expression when compared to controls. Images were taken using a Olympus BX51 microscope (magnification, 20×). Positive cells (brown) were quantitated using The Image-Pro plus v6.3 software package (Media Cybernetics, Inc.).

TNF-α and IL-6 are pro-inflammatory cytokines, and are considered as major biomarkers of inflammation. Groups 1, 2 and 3 mice together with their controls were sacrificed at the end of each treatment protocol; blood was collected via cardiac puncture. The serum obtained was evaluated for the levels of TNF-α and IL-6 using an ELISA kit. Both, TNF-α and IL-6 levels increased from Group 1 to Group 3 corresponding to increasing age and stages of cancer progression (Fig. 3A). UA-treatment significantly decreased serum TNF-α in Group 2 (by ∼90%) Group 3 (∼30%) (Fig. 3A, i) as well as serum IL-6 in all three groups by ∼40–90% (Fig. 3A, ii).

### UA suppresses the expression of cyclin D1, COX-2 and Caspase-3 in DLP of TRAMP mice

We next investigated various gene products involved in cancer progression (COX-2), proliferation (cyclin D1) and apoptosis (caspase-3) by immunohistochemistry in the DLP of Group 3 TRAMP mice. As shown in Fig. 3B, UA-treatment reduced the number of cells stained positive for cyclin D1 from 31% to 3% and COX-2 from 89% to 27%. As expected, the expression of caspase-3 increased from 6% to 95%. These results clearly indicated that inhibition of multiple gene products involved in tumor progression and the induction of apoptosis contribute to the potent anti-tumor activities of UA as observed in TRAMP tumor tissues.

### UA suppresses tumor growth and increases the survival of TRAMP mice

**Figure 4:**
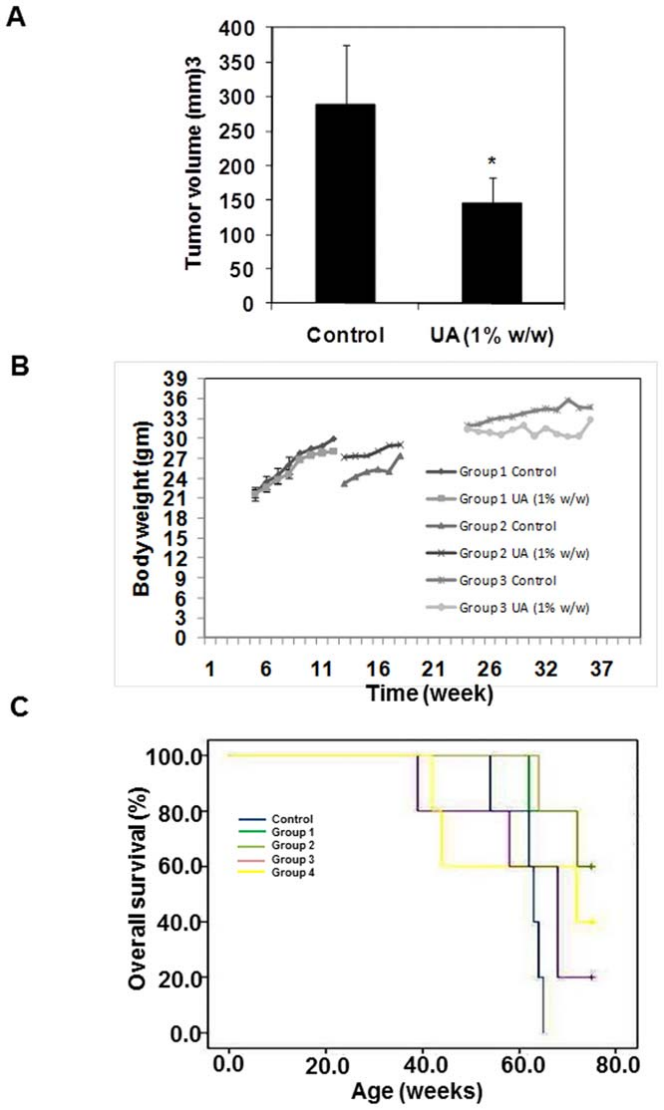
UA inhibits tumor growth in Group 3 TRAMP mice. **A,** Mice were euthanized at 36 weeks and the tumor volume was measured as described in Material and Methods. Error bars represent SEM. * Statistical significance (P<0.05). **B,** Body weight of Groups 1–3 TRAMP mice and their controls. **C,** The effect of UA-treatment on the long term survival of TRAMP mice. Kaplan-Meier survival analysis was performed for the control and UA-treated TRAMP mice. Median survival time was 55 weeks for control (n = 5). Median survival time for group 1, group 2, group 3 and group 4 was 75, 75, 68, and 72 weeks, respectively. Overall significance was determined using log-rank (Mantel-Cox) test. UA enriched diet significantly prolonged survival.

To determine if UA can suppress the growth of established prostate cancer, 24-week-old TRAMP-mice were fed with a control or UA (1%, w/w) enriched diet for 12 weeks and then sacrificed at the 36 weeks (Fig. 1A, Group 3). The wet weight of the prostate with seminal vesicles showed significant differences in the weight of the whole prostate gland. A significant (P<0.05) difference in the tumor volume was observed in the UA-treated mice (Fig. 4A). We also found that the long term feeding of UA enriched diet is safe without any organ or tissue toxicity with minimal effect on body weight (Fig. 4B). Throughout the 36 weeks period, age matched non-transgenic C57BL/6 mice was used as a negative controls, which showed none of the features associated with prostate cancer observed in TRAMP mice. All data presented on the effects of UA-treatment in TRAMP mice (Groups 1 to 3) are consistent with the survival data indicating that such treatment can significantly prolong the life span of mice (Fig. 4C). In addition to Groups 1, 2 and 3, a 4th group of TRAMP-mice were fed with the UA enriched diet for 36 weeks from 4 weeks of age (Group 4); control groups received normal diet (Fig. 1A). Mice were scored at the time of death based on tumor burden. Kaplan-Meier survival plots were generated to determine the overall survival. Log-rank (Mantel-Cox) plots showed increases in the overall survival of mice in all UA-treated groups. Median survival of mice in the control group was 55 weeks, compared to 75 weeks for Group 1 and Group 2, and 72 weeks for Group 4 (P<0.05). In group 3, the median survival was 68 weeks but failed to reach statistical significance (Fig. 4C). Therefore, it may be concluded that UA-treatment is most effective when it was commenced early when the TRAMP-mice showed no cancer or only early stages of PIN development.

### Detection of UA in serum

**Figure 5:**
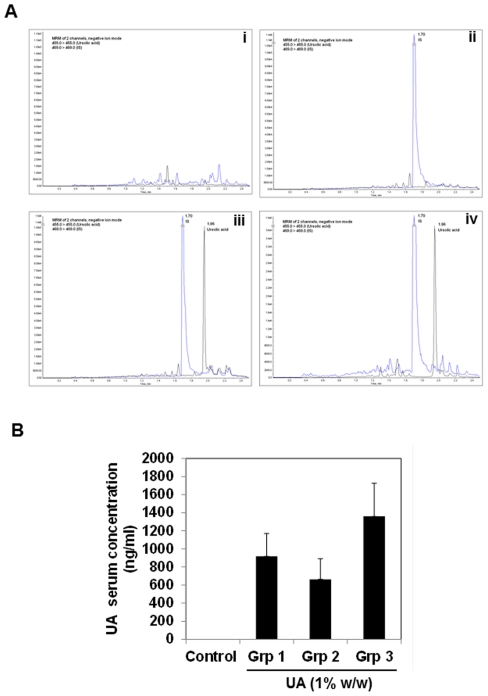
Mass spectrometric analysis of UA in serum. **A,** (i) Ion chromatograms of serum extracts from mice that had not received UA (blank). (ii) Full scan spectrum showing the m/z peak with a molecular mass of 469 corresponds to internal standard (IS) glycyrrhetinic acid. (iii) Full scan spectrum showing the m/z peak with a molecular mass of standard UA at 455 and IS at 469 and (iv) Full scan spectrum of sample, retention time was 1.7 and 1.96 min respectively for both IS and sample. The chromatograms are representative of 3 independent experiments. **B,** Serum concentrations of UA was calculated using Analyst software 1.4.2 from the linear regression equation of the peak area ratio against the concentration of the calibration curve. Error bars represent SEM.

UA was detected in serum of all 3 groups of TRAMP-mice fed with UA enriched diet. The concentrations detected varied from about 600 to 1300 ng/ml with no statistical significance between groups (Fig. 5B). As UA serum levels were expected to have reached steady state concentration in all three groups, the differences in concentration thus reflected individual variations. No additional peaks were detected in the serum, indicating that UA does not generate any metabolites (Fig. 5A, iii and iv). Our study presents the first experimental evidence that UA is well absorbed after oral feeding and the serum concentrations attained are sufficient to elicit biological effects as evident by the inhibition of prostate tumor growth in TRAMP mice.

## Discussion

The TRAMP mouse model is a well established spontaneously developing prostate cancer model that mimics natural prostate tumor progression, from PIN to highly invasive metastatic prostate cancer [20] in humans. In TRAMP mice, tumor originates from both DLP and ventral prostate (VP) as a result of Tag expression that is primarily restricted to the prostatic lobes [22], while human prostate cancer originates from the peripheral zone that is similar to the DLP in mice [23]. The anticancer effects of various chemopreventive agents, including oleanolic acid analogs, sulforaphane, polyphenols obtained from green tea, garlic constituents, curcumin, 3, 3′-diindolylmethane and genistein have been evaluated previously on spontaneously developing prostate tumorigenesis in TRAMP mice [24], [25], [26]. In addition, lycopene, γ-tocopherol, silbinin and tomato enriched diet have all been found to increase survival and delay the progression from PIN to adenocarcinoma and decrease the incidence of poorly differentiated carcinoma [23], [27], [28], [29], [30]. However, chemoprevention study with UA in prostate cancer model has not been carried out previously, and only our group has recently reported for the first time that UA enriched diet can indeed inhibit prostate tumor metastasis by modulating the CXCR4/CXCL12 axis in TRAMP mice [19]; although the effect of UA on tumor development and progression was not evaluated. In the present study, we studied the effect of UA treatment at three different stages of cancer development in TRAMP mice. We found that UA can significantly inhibit cancer development and progression at all three stages. In Group 1 mice, we observed measurable suppressive effects of UA on the PIN development, while in Group 2 mice, development of high grade PIN was inhibited as evidenced by the reduction in well differentiated PIN. Moreover, UA significantly inhibited the progression of preneoplastic PIN in DLP and tumor volume with no observable adverse effects in the Group 3 mice. The development of an abundance of non-cancerous lesions in this group as opposed to the predominance of cancerous lesions in control TRAMP mice is evident. Results of histological grading further suggested that UA can predominantly inhibit the development of precancerous lesions in a majority of mice, and thus prevent the development of highly invasive malignant lesions. UA also significantly increased overall survival by as much as 20 weeks, delayed progression from PIN to adenocarcinoma, and decreased the incidence of PDC.

Furthermore, we observed that the anti-proliferative and pro-apoptotic effects of UA are associated with the suppression of cyclin D1 and COX-2 and the inhibition of NF-κB, STAT3, AKT and IKKα/β activation in the prostates of TRAMP mice. Such inhibition correlated with the length of feeding period as evident by substantially greater inhibition in Group 3 mice, which received UA continuously for12 consecutive weeks. The reduction in TNF-α and IL-6 levels also correlated with the length of treatment period. The suppression of cyclin D1 and COX-2 may well be a consequence of the reduced activation of NF-κB and STAT3 as these pro-inflammatory transcription factors regulate the expression of cyclin D1 and COX-2 [2], [3], [31]. Increased caspase-3 expression is indicative of apoptosis. Taken together, these results clearly suggest that inhibition of cell proliferation and increase in apoptosis play a pivotal role in UA-mediated inhibition of prostate tumor progression in TRAMP mice. The fact that UA was detected in all serum samples in significant concentrations further indicate that UA is well absorbed and oral feeding can be considered as a preferred route of administration for a potential chemopreventive and therapeutic agent for prostate cancer. Therefore, it may be concluded that UA-treatment inhibited the progression of PIN to adenocarcinoma, reduced tumor growth and also prolonged the survival of mice. Together, these results indicate that UA may have a tremendous potential for use as a chemopreventive agent as well as a promising agent for prostate cancer treatment.

In summary, our data clearly demonstrate that UA feeding delays the progression of prostate cancer in mice through the modulation of various pro-inflammatory molecules. Because TRAMP is a spontaneous model for prostate cancer that closely mimics progressive forms of human disease, the results of this study provide a sound basis for pursuing further studies on the use of UA either alone or in combination with other therapy for the prevention and/or treatment of prostate cancer.

## Materials and Methods

### Reagents

UA (98% pure) was purchased from Guangxi Changzhou Natural Products Development Company Ltd (China). UA was mixed with normal mouse diet at 1% w/w and stored at 4°C. Tris, glycine, NaCl, SDS, glycyrrhetinic acid and β-actin antibody were purchased from Sigma-Aldrich (St. Louis, MO, USA) and HRP-conjugated secondary antibody was obtained from Invitrogen (Carlsbad, CA, USA). Antibodies against phospho-STAT3 (Tyr 705), phospho-AKT (Ser 473), STAT3 were purchased from Santa Cruz Biotechnology (Santa Cruz, CA). Antibodies to AKT, p65, phospho-specific IKKα/β (Ser 180/Ser 181), and IKKα were purchased from Cell Signaling Technology (Beverly, MA). TNF-α and IL-6 ELISA kits were purchased from Research Instruments (MA, USA). Bradford reagent was purchased from Bio-Rad (Hercules, CA). Immunohistochemistry staining kit was purchased from Dako (Denmark).

### TRAMP mice breeding and genotyping

Animal experiments were conducted in accordance with Singapore NACLAR guidelines (Law as of November 2004) for laboratory animal use and care and approved by NUS Institutional Animal care and Use Committee protocol number 052/09. Briefly, 4-week-old TRAMP female mice purchased from The Jackson Laboratory were mated with C57BL/6 male at NUS CARE (Singapore). The pups were genotyped for the transgene (Tag) at 3 weeks of age; DNA was extracted from the tail snips using TRIZOL® reagent (Invitrogen, USA) and quantified using Nanodrop spectrophotometer (Thermo scientific, USA). One µg of the DNA was taken for PCR analysis. Polymerase chain reaction (PCR) reaction mixture contained 10 µl of 5× QIAGEN PCR buffer, 1 µg of DNA, 0.6 µM each of forward and reverse primers, 2 µl of dNTP mix and 1 µl of Taq polymerase enzyme in a final volume of 50 µl. Primers used for genotyping, forward primer: 5′-CCG GTC GAC CGG AAG CTT CCA CAA GTG CAT TTA-3′, Reverse primer: 5′-AGG CAT TCC ACC ACT GCT CCC ATT CAT C-3′. PCR conditions were 94°C for 3 min, 94°C for 30 sec, 62°C for 1 min, 72°C for 1 min, repeat steps 2–4 for 35 cycles, 72°C for 2 min and held at 4°C. PCR products were run on 1% agarose gel containing 1× GelRed nucleic acid gel stain from Biotium (Hayward, CA). Stained bands were visualized under UV light and photographed using Biorad Gel doc EZ system (Bio-Rad, USA). Transgene positive pups were then used for the subsequent experiments.

### Pathological grading of tumor tissues

The pathological grading of TRAMP prostate cancer was performed according to the grading system described previously [22]. Prostate lesions in the DLP were histologically graded as normal (duct lined with single layer of secretory epithelial cells surrounded by two or three cell layers of fibrousmuscular stroma), low-grade prostatic intraepithelial neoplasia (PIN) [epithelial cells with variably elongated nuclei with condensed chromatin], high-grade PIN (epithelial stratification and tufting, presence of micropapillary and cribiform structures), well-differentiated carcinoma (WDC) (epithelial cells invading fibromuscular stroma), and moderately differentiated (MDC) to poorly differentiated (PDC) adenocarcinoma of the prostate (sheets of neoplastic cells with little or no glandular structures). 15 randomly selected microscopic fields on hematoxylin and eosin (H&E) stained sections of the DLP were scored for the incidence and pathological grade of the prostate cancer in control and UA fed TRAMP mice.

### *In vivo* anti-tumor study

The inbred male TRAMP mice, 4 weeks old, were maintained in temperature controlled rooms with a 12 h light/dark cycle. All mice were weighed before start of experiment. The mice were then randomized into the following UA fed and control groups (n = 5). Group 1: 4 weeks old TRAMP mice were fed with UA (1% w/w) enriched diet for 8 weeks; Group 2: 12 weeks old TRAMP mice were fed with UA enriched diet for 6 weeks; Group 3: 24 weeks old TRAMP mice were fed with UA enriched diet for 12 weeks. Control TRAMP mice received normal diet. Body weight and tumor size were recorded twice every week and the tumor size was determined by Vernier caliper and calculated using the formula [length×(width)^2^]/2. Mice were euthanized by CO_2_ inhalation followed by cervical dislocation. Blood samples, collected by cardiac puncture, were kept at 4°C overnight and centrifuged at 10,000 rpm for 20 min to obtain serum, which was stored in aliquots at −80°C. Tumor volume was measured and weighed. Prostate gland was microdisected from the seminal vesicles under a stereomicroscope, fixed in 10% phosphate buffered formalin for H&E and immunohistochemical analysis.

### ELISA assay for TNF-α and IL-6

Stored serum samples were analyzed for TNF-α and IL-6 levels using ELISA kit (R&D systems, USA) according to manufacturer instructions. Sandwich ELISA protocols were used and calibration was done with standard TNF-α and IL-6 provided in the kit.

### Western blotting

Dorsolateral prostate tumor tissues were lysed in lysis buffer containing (20 mM Tris (pH 7.4), 250 mM NaCl, 2 mM EDTA (pH 8.0), 0.1% Triton X-100, 0.01 mg/ml aprotinin, 0.005 mg/ml leupeptin, 1 mM PMSF, and 4 mM NaVO4). Lysates were then centrifuged at 13,000 rpm for 10 min to remove insoluble material and 50 µg of protein was resolved on a 10% SDS gel. After electrophoresis, proteins were electrotransferred to a nitrocellulose membrane, blocked with 5% nonfat milk, and probed with antibodies of interest overnight at 4°C. The blot was washed, exposed to HRP-conjugated secondary antibodies for 1 h, and finally examined by chemiluminescence (ECL; GE Healthcare, Little Chalfont, Buckinghamshire, UK). Densitometric analysis of the scanned blots was performed using Image J software and the results are expressed as fold change relative to control.

### Immunohistochemistry

DLP tumor tissues were fixed with 10% phosphate buffered formalin, processed and embedded in paraffin. Tissue sections, 5 µm, were cut and deparafinized as described previously [18]. Images were taken using a Olympus BX51 microscope (magnification, 20×). Positive cells (brown) were quantitated using The Image-Pro plus v6.3 software package (Media Cybernetics, Inc.).

### Survival studies

To investigate whether UA enriched diet can prolong the life span of TRAMP mice, 25 male inbred TRAMP male mice were divided into control and UA fed groups (n = 5). Group 1: 4 weeks old TRAMP mice were fed with UA enriched diet for 8 weeks; Group 2: 12 weeks old TRAMP mice were fed with UA enriched diet for 6 weeks; Group 3: 24 weeks old TRAMP mice were fed with UA enriched diet for 12 weeks; Group 4: 4 week old mice were fed with UA enriched diet for 36 weeks. Control TRAMP mice received normal diet. Animals were monitored weekly for body weight and tumor development by abdominal pelvic palpation and survival.

### Determination of UA in serum samples

Extraction of UA from mice serum samples were carried out by liquid-liquid extraction (LLE) as previously described [32]. UA concentrations in mice serum samples were then determined using ultra performance liquid chromatography-mass spectrometry (UPLC-MS) as previously described with modifications [33]. Mass spectrometry data acquisition and subsequent analyses were carried out using linear ion trap quadrupole mass spectrometer (3200 QTRAP) equipped with a TurboIonSpray electrospray ionization (ESI) source and Analyst 1.4.2 software (Applied Biosystems, Foster City, CA, USA). Quantitation of analyte and internal standard (IS) was done using single ion monitoring in multiple reactions monitoring mode (MRM, *m/z* 455.0 and *m/z* 469.0 were used for UA and IS, respectively) (18). Representative chromatograms are shown in Fig. 5A. The serum concentration of UA was calculated using Analyst software 1.4.2 (Applied Biosystem, USA).

### Statistical analysis

Experiments were carried out in triplicates and repeated twice. The significance of differences between groups was evaluated by Student's t-test and a p value of less than 0.05 was considered statistically significant. Kaplan-Meier survival plots were generated to determine the survival of mice and log-rank (Mantel-Cox) test was performed to determine the significance difference between control and UA fed groups.
